# PD-L1 expression evaluated by 22C3 antibody is a better prognostic marker than SP142/SP263 antibodies in breast cancer patients after resection

**DOI:** 10.1038/s41598-021-97250-2

**Published:** 2021-10-01

**Authors:** Yoon Jin Cha, Dooreh Kim, Soong June Bae, Sung Gwe Ahn, Joon Jeong, Hye Sun Lee, Soyoung Jeon, Tae-Kyung Yoo, Woo-Chan Park, Chang Ik Yoon

**Affiliations:** 1 Department of Pathology, Gangnam Severance Hospital, Yonsei University College of Medicine, Seoul, Korea; 2 Institute for Breast Cancer Precision Medicine, Yonsei University College of Medicine, Seoul, Korea; 3 Division of Breast Surgery, Department of Surgery, Seoul St Mary’s Hospital, College of Medicine, The Catholic University of Korea, 222 Banpo-daero, Seocho-gu, Seoul, Korea; 4 Department of Surgery, Gangnam Severance Hospital, Yonsei University College of Medicine, Seoul, Korea; 5 Biostatistics Collaboration Unit, Yonsei University College of Medicine, Seoul, Korea

**Keywords:** Breast cancer, Surgical oncology, Tumour biomarkers

## Abstract

Immune checkpoint inhibitors (ICI) have demonstrated efficacy in the treatment of solid cancers. However, there is no unified predictive biomarker available for ICIs. We aimed to compare the prognostic impact of using three PD-L1 antibodies (SP142, SP263, and 22C3) for immunohistochemical (IHC) analysis. We retrospectively investigated tumor tissues derived from 316 breast cancer cases, by constructing tissue microarrays and by performing IHC staining. The immune-cell expression rate (for SP142 and SP263) and combined proportional score (for 22C3) were evaluated, and survival outcomes were analyzed. Prediction models were developed, and values of Harrel’s c-index and areas under curves were calculated to compare the discriminatory power. Negative PD-L1 expression based on the 22C3-IHC assay was determined to be an independent prognostic marker for recurrence-free survival (RFS, *P* = 0.0337) and distant metastasis-free survival (DMFS, *P* = 0.0131). However, PD-L1 expression based on SP142- and SP263-IHC assays did not reveal a prognostic impact. Among the three antibodies, adding PD-L1 expression data obtained via 22C3-IHC assay to the null model led to a significant improvement in the discriminatory power of RFS and DMFS. We suggest that PD-L1 expression based on the 22C3-IHC assay is a superior prognostic marker than that based on SP142- and SP263-IHC assays.

## Introduction

Immunotherapy targeting programmed cell death protein 1 (PD-1) or programmed death ligand 1 (PD-L1) has demonstrated remarkable efficacy in the treatment of several solid cancers including breast cancer^1–8^. PD-1, a transmembrane protein, plays an important role in downregulating functions of the immune system^9^. PD-L1 can be expressed in both tumor and immune cells. PD-L1/PD-1 binding has been shown to induce immune tolerance in peripheral tissues^9,10^, in which the PD-L1/PD-1 signaling pathway promotes tumor escape from immune surveillance^10,11^. Currently, diagnostic factors used to predict survival outcomes include the expression profiles of the tumor micro-environment and PD-L1/co-inhibitory proteins^12,13^. PD-L1 immunohistochemistry (IHC) is the only clinically approved method for predicting response to immune checkpoint inhibitor therapy. However, PD-L1 IHC testing is complex due to the availability of multiple IHC assays, each with its own reagents and other clinical designs^4,5,8^, resulting in nonuniform clinical application of PD-L1 IHC.

This study aimed to perform a comprehensive, retrospective evaluation of PD-L1 expression based on IHC assays with three PD-L1 antibodies (SP142, SP263, and 22C3) in tumors of patients with breast cancer. Accordingly, we investigated PD-L1 expression in tumor samples and compared survival outcomes predicted by using PD-L1 expression data obtained using each PD-L1 antibody.

## Results

### Patient characteristics based on PD-L1 expression

A total of 316 breast cancer patients at Gangnam Severance Hospital were included in this study. The median patient age was 50 years (range 25–86 years). Clinical characteristics were grouped and compared based on PD-L1 expression according to antibody-IHC assays, as shown in Table 1. Positive PD-L1 expression was associated with higher Ki67, estrogen receptor (ER) and progesterone receptor (PR) negativity, triple-negative breast cancer (TNBC) subtype, and higher histological grade (HG). Positive PD-L1 expression was related to lower American Joint Committee on Cancer (AJCC) stage for the SP142 group, but not for the SP263 or 22C3 groups. Positive PD-L1 expression was associated with the receipt of chemotherapy for the SP263 group. There was no statistical difference in age, lympho-vascular invasion (LVI), and receipt of radiotherapy between the three groups.

**Table 1:** Comparison of patient and tumor characteristics, and PD-L1 status in patients with breast cancer.

	22C3			SP142			SP263		
	Negative, n = 232	Positive, n = 57	*P-value*	Negative, n = 258	Positive, n = 43	*P-value*	Negative, n = 184	Positive, n = 115	*P-value*
	N (%)	N (%)		N (%)	N (%)		N (%)	N (%)	
Age (year, mean ± SD)	50.3 ± 11.2	49.4 ± 11.2	0.575	50.6 ± 11.2	49.2 ± 12.8	0.445	50.9 ± 11.2	49.9 ± 11.6	0.441
Ki67 (%, mean ± SD)	20.9 ± 18.9	41.3 ± 21.9	< 0.001	23.5 ± 21.0	34.6 ± 20.1	0.002	19.9 ± 19.1	34.2 ± 21.6	< 0.001
**ER**			< 0.001			0.008			< 0.001
Positive	84 (36.2)	3 (5.3)		81 (31.4)	5 (11.6)		74 (40.2)	12 (10.4)	
Negative	148 (63.8)	54 (94.7)		177 (68.6)	38 (88.4)		110 (59.8)	103 (89.6)	
**PR**			< 0.001			0.003			< 0.001
Positive	73 (31.5)	2 (3.5)		72 (27.9)	3 (7.0)		66 (35.9)	9 (7.8)	
Negative	159 (68.5)	55 (96.5)		186 (72.1)	40 (93.0)		118 (64.1)	106 (92.2)	
**HER2**^**a**^			0.427			0.503			0.642
Positive	51 (22.0)	10 (17.5)		54 (20.9)	7 (16.3)		39 (21.2)	22 (19.1)	
Negative	177 (76.3)	47 (82.5)		201 (77.9)	35 (81.4)		142 (77.2)	92 (80.0)	
Missing	4 (1.7)	0		3 (1.2)	1 (2.3)		3 (1.6)	1 (0.9)	
**Subtype **^**a**^			< 0.001			0.001			< 0.001
Luminal/HER2(-)	71 (30.6)	1 (1.8)		70 (27.1)	1 (2.3)		64 (34.8)	7 (6.1)	
HER2 ( +)	49 (21.1)	10 (17.5)		52 (20.2)	7 (16.3)		37 (20.1)	22 (19.1)	
TNBC	108 (46.6)	46 (80.7)		133 (51.6)	34 (79.1)		80 (43.5)	85 (73.9)	
Missing	4	0		3 (1.2)	1 (2.3)		3 (1.6)	1 (0.9)	
**HG**^**a**^			< 0.001			< 0.001			< 0.001
I, II	119 (51.3)	12 (21.1)		126 (48.8)	9 (20.9)		107 (58.2)	28 (24.3)	
III	106 (45.7)	45 (78.9)		123 (47.7)	34 (79.1)		71 (38.6)	84 (73.0)	
Missing	7 (3.0)	0		9 (3.5)	0		6 (3.3)	3 (2.6)	
**AJCC stage**^**#, a**^			0.181			0.042			0.287
I	62 (26.7)	21 (36.8)		70 (27.1)	20 (46.5)		52 (28.3)	40 (34.8)	
II	128 (55.2)	33 (57.9)		149 (57.8)	19 (44.2)		102 (55.4)	61 (53.0)	
III	30 (12.9)	3 (5.3)		29 (11.2)	2 (4.7)		21 (11.4)	11 (9.6)	
Missing	12 (5.2)	0		10 (3.9)	2 (4.7)		9 (4.9)	3 (2.6)	
**LVI**^**a**^			0.194			0.575			0.157
Negative	164 (70.7)	47 (82.5)		41 (15.9)	5 (11.6)		33 (17.9)	13 (11.3)	
Positive	43 (18.5)	7 (12.3)		191 (74.0)	31 (72.1)		134 (72.8)	87 ((75.7)	
Missing	25 (10.8)	3 (5.3)		26 (10.1)	7 (16.3)		17 (9.2)	15 (13.0)	
**Chemotherapy**^**a**^			0.125			0.779			0.043
Done	191 (82.3)	52 (91.2)		216 (83.7)	37 (86.0)		147 (80.0)	103 (89.6)	
Undone	39 (16.8)	5 (8.8)		40 (15.5)	6 (14.0)		35 (19.0)	12 (10.4)	
Missing	2 (0.9)	0		2 (0.8)	0		2 (1.1)	0	
**Neoadjuvant chemotherapy**^**a**^			0.789			0.355			0.109
Done	4 (1.7)	0		5 (1.9)	0		4 (2.2)	0	
Undone	226 (97.4)	57 (100)		251 (97.3)	43 (100)		178 (96.7)	115 (100)	
Missing	2 (0.9)	0		2 (0.8)	0		2 (1.1)	0	
**Radiotherapy**^**a**^			0.104			0.945			0.317
Done	98 (42.2)	31 (54.4)		121 (46.9)	20 (46.5)		83 (45.1)	59 (51.3)	
Undone	133 (57.3)	26 (45.6)		136 (52.7)	23 (53.5)		100 (54.3)	56 (48.7)	
Missing	1 (0.4)	0		1 (0.4)	0		1 (0.5)	0	
**Endocrine therapy**			< 0.001			0.017			< 0.001
Done	107 (46.1)	5 (8.8)		103 (39.9)	9 (20.9)		95 (51.6)	17 (14.8)	
Undone	125 (53.9)	52 (91.2)		155 (60.1)	34 (79.1)		89 (48.4)	98 (85.2)	

SD, standard deviation; ER, estrogen receptor; PR, progesterone receptor; HER-2, human epidermal growth factor receptor-2; TNBC, triple negative breast cancer; HG, histological grade; LVI, lympho-vascular invasion.

^a^Percentages calculated without missing values.

### Correlation of PD-L1 assays with breast cancer tissue microarray (TMA) results

**Figure 1:**
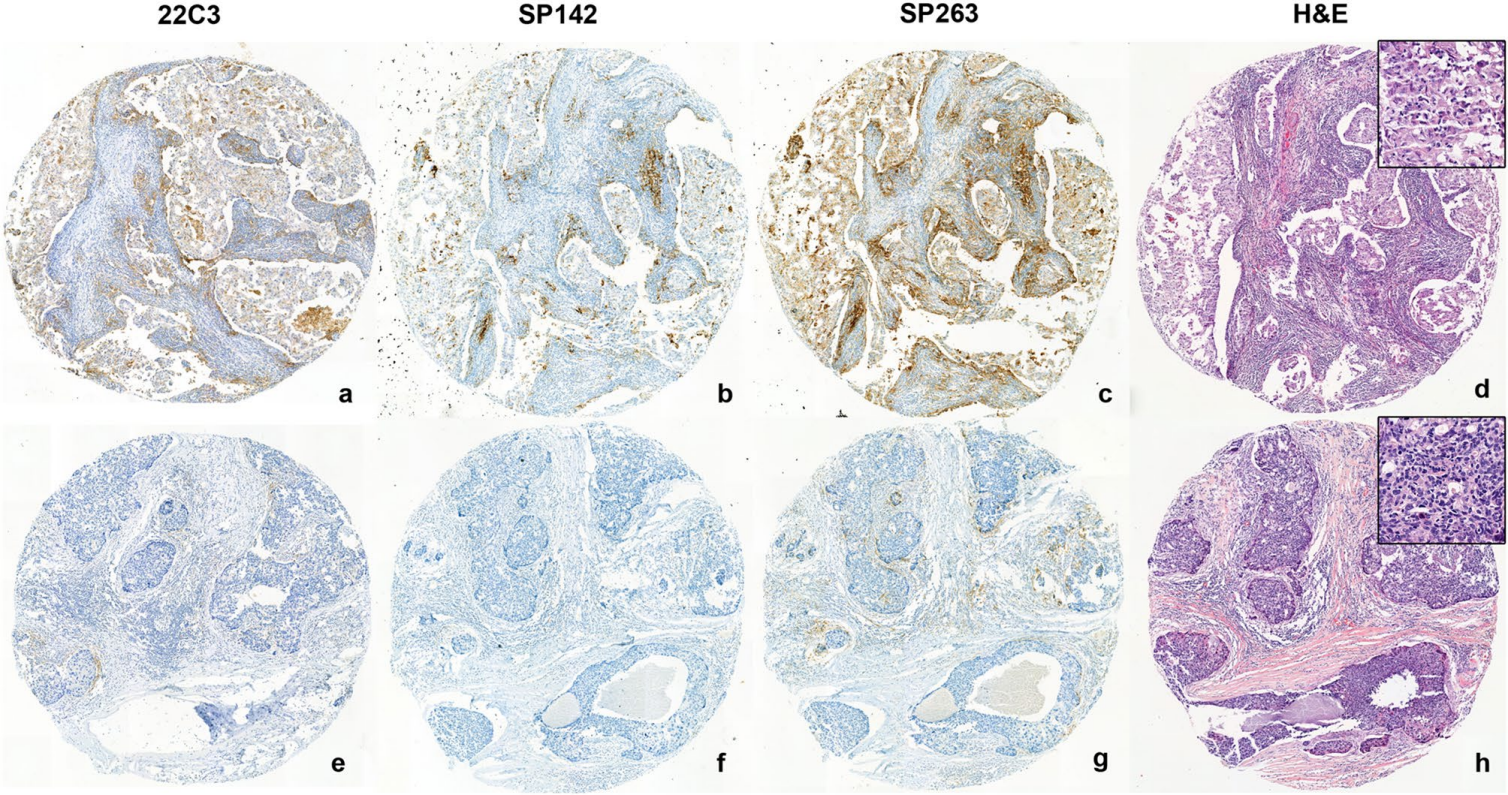
Positive and negative expression patterns of three PD-L1 antibodies. A representative case that showed triple-positive features for all three antibodies (**a**–**d**) exhibited > 90% of the stromal tumor-infiltrating lymphocytes (TIL) infiltration as well as intratumoral TIL. Discordant case which was positive for 22C3 and SP263 antibodies but lacked SP142 expression (**e**–**h**) presented with less TIL infiltration than that shown by the triple-positive case.

**Figure 2:**
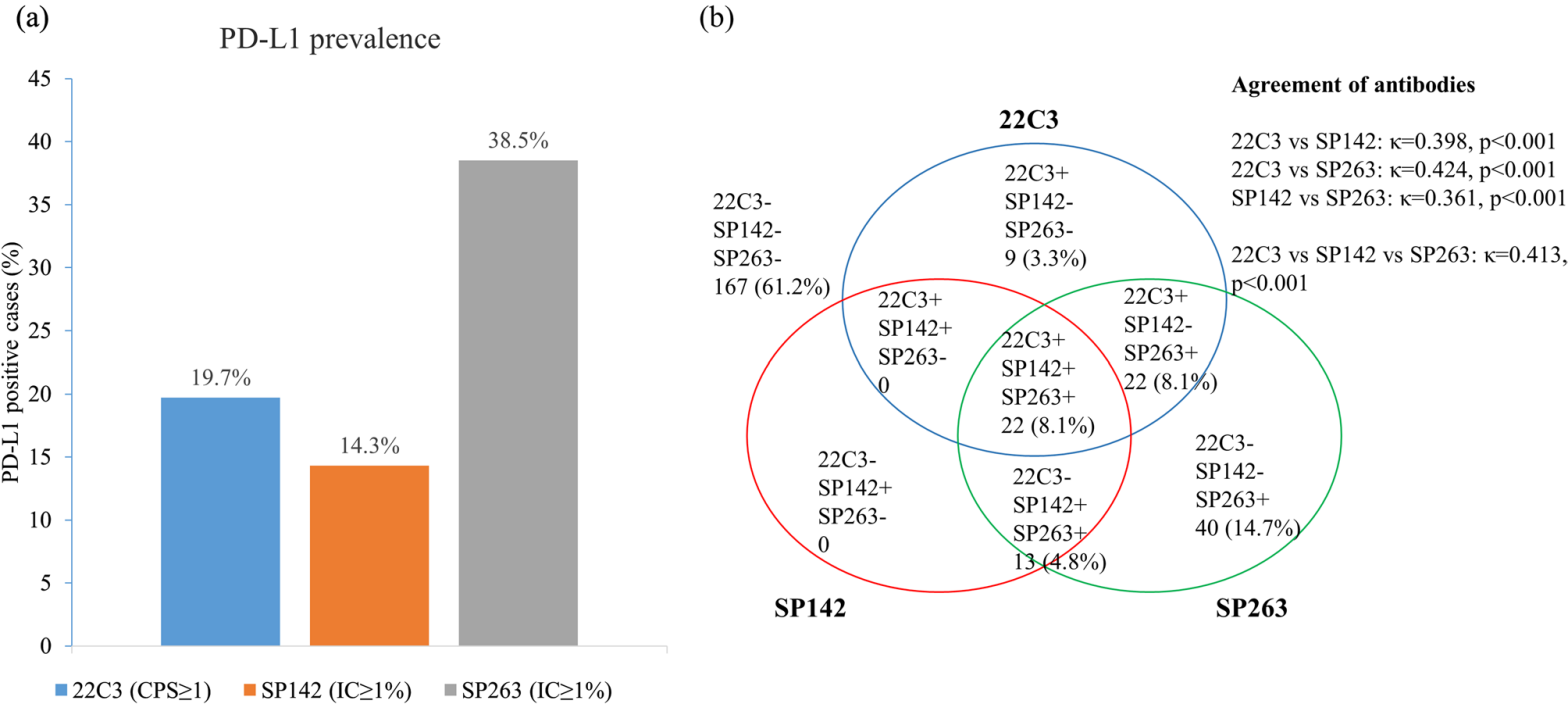
Immunohistochemical staining patterns of PD-L1 based on the use of three PD-L1 antibodies: (**a**) 57/289 patients (19.7%) presented with positive PD-L1-expressing tumors using 22C3; 43/301 patients (14.3%) presented with positive PD-L1-expressing tumors using SP142; 115/299 patients (38.5%) presented with positive PD-L1-expressing tumors using SP263. (**b**) Venn diagram illustrated for correspondence and Kappa value of comparison of PD-L1 staining using three PD-L1 antibodies.

TMAs with tumor tissues derived from 316 patients were subjected to staining procedures for each antibody. The representative case that was triple-positive for all three antibodies (Fig. 1a–d) presented with > 90% of stromal tumor-infiltrating lymphocytes (TIL) infiltration as well as intratumoral TIL. The discordant case that was positive for 22C3 and SP263 antibodies, but lacked SP142 expression (Fig. 1e–h), exhibited lesser extent of TIL infiltration than the triple-positive case. For IHC assays, 289/316 (91.5%) of the investigated TMA cores were subjected to staining procedures and their PD-L1 expression was studied using the 22C3 antibody, whereas the PD-L1 expression of 301/316 (95.3%) and 299/316 (94.6%) of the TMA cores was studied using SP142 and SP263 antibodies, respectively. The prevalence of positive PD-L1 expression differed according to each PD-L1 antibody. Based on the results obtained with the use of the 22C3 antibody, 19.7% of the patients with breast cancer harbored PD-L1-positive tumors [combined positive score (CPS) ≥ 1]. However, only 14.3% of the patients harbored PD-L1-positive tumors according to SP142 staining (≥ 1% IC), and PD-L1 IHC staining using SP263 led to the obtainment of a higher numbers of patients with PD-L1-positive tumors (38.5%) (Fig. 2a). Tumor tissues derived from 273 patients were subjected to staining procedures with all three PD-L1 antibodies, and the correspondence among PD-L1 expression in the three groups has been depicted in Fig. 2b. Agreement between the findings obtained using the three antibodies was moderate (κ = 0.413, *P* < 0.001). There was moderate agreement between 22C3 and SP263 (κ = 0.424, *P* < 0.001), but 22C3 vs. SP142 (κ = 0.398, *P* < 0.001), and SP142 vs. SP263 (κ = 0.361, *P* < 0.001) showed fair agreements.

### Prognostic significance of PD-L1 expression based on the antibodies

**Figure 3:**
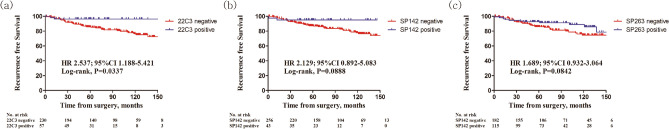
Kaplan–Meier survival curves of recurrence-free survival (RFS) in relation to PD-L1 expression based on PD-L1 antibody-IHC assays in patients with breast cancer. (**a**) Patients with negative PD-L1 expression based on the 22C3-IHC assay were associated with poor RFS (HR 2.537, 95% CI 1.188–5.421, *P* = 0.0337, log-rank test). (**b**, **c**) PD-L1 expression with SP142- and SP263-IHC assays did not show significantly different RFS.

**Figure 4:**
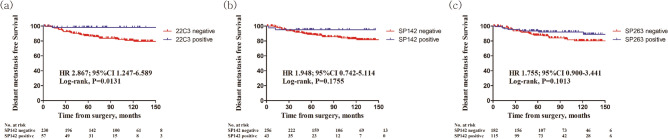
Kaplan–Meier survival curves of distant metastasis-free survival (DMFS) in relation to PD-L1 expression based on PD-L1 antibody-IHC assays in patients with breast cancer. (**a**) Patients with negative PD-L1 expression based on the 22C3-IHC assay were associated with poor DMFS (HR 2.867, 95% CI 1.247–6.589, *P* = 0.0131, log-rank test). (**b**, **c**) DMFS did not significantly differ based on SP142- and SP263-IHC assay results.

At a median follow-up time of 78.5 months (range, 0–325 months), 49 patients developed recurrence. Among them, 39 exhibited distant metastasis and 14 demonstrated loco-regional recurrence (four cases presented with distant metastasis and loco-regional recurrence simultaneously). There were 2, 9, and 10 recurrences in cases of discrepancy between 22C3 and SP142, 22C3 and SP263, and SP142 and SP263, respectively. Only negative PD-L1 expression based on the 22C3-IHC assay was significantly associated with decreased recurrence-free survival (RFS) [Fig. 3a; hazard ratio (HR) 2.537, 95% confidence intervals (CI) 1.188–5.421, *P* = 0.0337] and distant metastasis-free survival (DMFS) (Fig. 4a; HR 2.867, 95% CI 1.247–6.589, *P* = 0.0131, log rank test). However, RFS and DMFS did not differ significantly between negative and positive PD-L1 expression levels based on SP142- and SP263-IHC assays (Figs. 3b,c, 4b,c).

In the univariate Cox proportional hazard model, age and positive PD-L1 expression based on the 22C3-IHC assays were found to be significant prognostic factors for RFS (Table 2, HR 0.207 95%, CI 0.050–0.855, *P* = 0.0295). However, PD-L1 expression based on SP142- or SP263-IHC assays was not a significant factor in RFS (Table 2). In the multivariate analysis, PD-L1 expression based on the 22C3-IHC assays was confirmed as a significant predictor for RFS (Table 2, HR 0.206, 95% CI 0.050–0.853, *P* = 0.0293).

**Table 2﻿:** Cox proportional hazard analysis for recurrence-free survival (RFS).

	Univariate analysis		Multivariate analysis	
	HR (95% CI)	*P-*value	HR (95% CI)	*P-*value
Age	0.972 (0.945–0.999)	0.0407	0.972 (0.945–0.999)	0.0401
**HG**		0.8568		
I, II	1			
III	0.947 (0.52–1.713)			
**LVI**		0.7601		
Negative	1			
Positive	1.136 (0.500–2.580)			
Ki67	0.994 (0.977–1.011)	0.472		
**ER**		0.977		
Negative	1			
Positive	0.991 (0.532–1.845)			
**PR**		0.794		
Negative	1			
Positive	0.917 (0.477–1.762)			
**HER2**		0.184		
Negative	1			
Positive	0.560 (0.238–1.318)			
**Tumor size**		0.4306		
≤ 2 cm	1			
> 2 cm	1.279 (0.694–2.356)			
**Lymph node metastasis**		0.464		
Negative	1			
Positive	1.248 (0.690–2.255)			
**Chemotherapy**		0.5169		
Not done	1			
Done	0.776 (0.361–1.669)			
**Radiotherapy**		0.2119		
Not done	1			
Done	1.454 (0.808–2.619)			
**22C3**		0.0295		0.0293
Negative	1		1	
Positive	0.207 (0.050–0.855)		0.206 (0.050–0.853)	
**SP142**		0.1692		
Negative	1			
Positive	0.370 (0.090–1.527)			
**SP263**		0.1445		
Negative	1			
Positive	0.603 (0.305–1.190)			

HR, hazard ratio; CI, confidence interval; HG, histologic grade; LVI, lympho-vascular invasion; ER, estrogen receptor; PR, progesterone receptor; HER-2, human epidermal growth factor receptor-2.

In the univariate analyses of DMFS, only PD-L1 expression based on the 22C3-IHC assay was a significant factor of favorable DMFS (Supplementary Table 1; HR 0.122, 95% CI 0.017–0.888, *P* = 0.0378). Multivariate analysis confirmed that PD-L1 expression based on the 22C3-IHC assay was significantly associated with favorable DMFS (Supplementary Table 1; HR 0.121, 95% CI 0.017–0.886, *P* = 0.0376).

### Evaluation of Cox proportional hazard model using Harrel’s c-index, net reclassification index (NRI), integrated discrimination improvement (IDI), and time dependent areas under the curve (AUC)

To quantify the improvement of predictive ability contributed by PD-L1 expression according to each antibody utilized, we calculated Harrel’s c-index, NRI, and IDI for the multivariate models. The addition of data on PD-L1 expression based on the 22C3-IHC assay to the null model significantly increased the *c*-index for both RFS (model 1 in Table 3; HR 0.626, 95% CI 0.536–0.689, *P* = 0.0001) and DMFS (model 1 in Supplementary Table 2; HR 0.633, 95% CI 0.574–0.692, *P* < 0.0001). Furthermore, the addition also improved the discriminatory power of RFS measured by considering NRI and IDI (model 1 in Table 3; *P* = 0.0008 and *P* = 0.04, respectively). Moreover, it also demonstrated superior discrimination of DMFS measured by considering NRI (model 1 in Supplementary Table 2, *P* < 0.0001) and IDI (model 1 in Supplementary Table 2, *P* = 0.044). However, addition of PD-L1 expression data based on SP142- or SP263-IHC assays did not substantially improve the discriminatory power of RFS and DMFS (model 2 and 3 in Table 3 and Supplementary Table 2).

**Table 3:** Evaluation of multivariate Cox proportional hazard model using Harrel’s *c*-index, NRI, IDI, and time-dependent AUC for RFS.

	Null model		Model 1		Model 2		Model 3	
	HR (95% CI)	*P*-value	HR (95% CI)	*P*-value	HR (95% CI)	*P*-value	HR (95% CI)	*P*-value
Harrel’s c index	0.584 (0.531–0.637)	0.0019	0.626(0.536–0.689)	0.0001	0.587(0.526–0.648)	0.0050	0.612(0.541–0.683)	0.0019
NRI	1		0.112(0.001–0.201)	0.0008	0.055(− 0.007–0.140)	0.0879	0.079(− 0.055–0.230)	0.2517
IDI	1		0.014(0.002–0.036)	0.04	0.003(− 0.002–0.022)	0.3436	0.007(− 0.001–0.036)	0.1938
Time dependent AUC	0.587 (0.544–0.630)	0.0001	0.636(0.585–0.687)	< 0.0001	0.606(0.557–0.655)	< 0.0001	0.606(0.543–0.669)	0.0009

AUC, area under the curve; CI, confidence interval; HR, hazard ratio; IDI, integrated discrimination improvement; NRI, net reclassification index; RFS, recurrence-free survival.

*Null model: including age.

**Model 1: Null model + 22C3.

***Model 2: Null model + SP142.

****Model 3: Null model + SP263.

**Figure 5:**
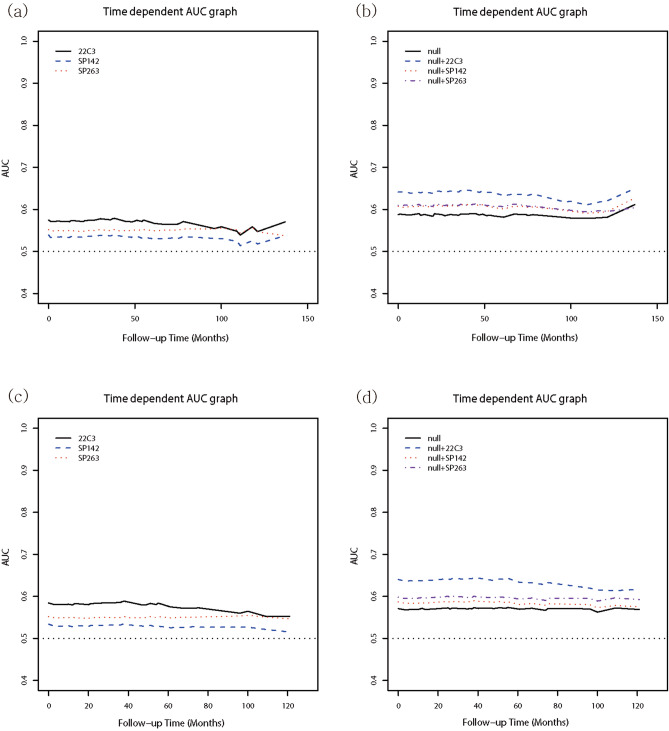
Comparison of improved discriminatory performance for each PD-L1 antibody using the time-dependent AUC graphs of RFS and DMFS. (**a**) Among the three PD-L1 antibodies, 22C3-IHC staining showed a higher AUC value in RFS. (**b**) Via addition to the null model in RFS, the improved AUC value based on the 22C3-IHC assay was observed to be superior among the three PD-L1 antibodies (AUC of 22C3 = 0.636, AUC of SP142 = 0.606, AUC of SP263 = 0.606). (**c**) Among the three PD-L1 antibodies, 22C3-IHC staining demonstrated a higher AUC value for DMFS. (d) Via addition to the null model in DMFS, the improved AUC value based on the 22C3-IHC assay was found to be superior among the three PD-L1 antibodies (AUC of 22C3 = 0.634, AUC of SP142 = 0.584, AUC of SP263 = 0.596).

In the time-dependent AUC model for RFS, the addition of PD-L1 expression data based on each of the three antibodies utilized to the null model increased the discriminatory ability (Table 3). However, addition of PD-L1 expression data based on the 22C3-IHC assay to the null model yielded higher discriminatory value than that based on SP142- and SP263-IHC assays (Table 3, Fig. 5a,b). In the time-dependent AUC model for DMFS, addition of PD-L1 expression data based on the 22C3-IHC assay to the null model demonstrated superior discriminatory power (Supplementary Table 2) than that based on SP142- or SP263-IHC assays (Supplementary Table 2 and Fig. 5c,d).

### Prognostic impact of PD-L1 expression in TNBC

Clinical characteristics were compared according to PD-L1 expression based on each PD-L1 antibody-IHC assay performed for the TNBC subtype, as shown in Supplementary Table 3. Positive PD-L1 expression using the SP263 antibody was associated with HG and higher Ki67, and negative PD-L1 expression using the SP142 antibody was related to lower AJCC stage. Otherwise, there was no statistical difference in the other clinicopathologic characteristics.

Survival outcomes based on the PD-L1 expression according to each antibody utilized were compared for the TNBC subtype. RFS differed significantly according to the PD-L1 expression in all antibody-IHC assays (Supplementary Fig. 1a; HR 3.462, 95% CIs 1.489–8.048, *P* = 0.0039; Supplementary Fig. 1b; HR 2.701, 95% CIs 1.026–7.108, *P* = 0.0442, Supplementary Fig. 1c; HR 2.371, 95% CIs 1.097–5.127, *P* = 0.0281, respectively). Additionally, decreased DMFS was observed in the negative 22C3- and SP263-IHC assays in the TNBC subtype (Supplementary Fig. 2a; HR 4.184, 95% CIs 1.710–10.24, *P* = 0.0017; Supplementary Fig. 2c; HR 2.746, 95% CIs 1.188–6.350, *P* = 0.0181, respectively), but it was not observed in the SP142-IHC assay (Supplementary Fig. 2b; HR 2.611, 95% CIs 0.922–7.398, *P* = 0.0708).

Univariate analysis of RFS in each PD-L1 antibody was performed using the Cox proportional hazard model. In the TNBC subtype, positive PD-L1 expression based on 22C3- and SP263-IHC assays was a significant prognostic factor for RFS (Supplementary Table 4; 22C3, HR 0.095, 95% CI 0.013–0.700, *P* = 0.021; SP263, HR 0.406, 95% CI 0.176–0.934, *P* = 0.0034, respectively). However, PD-L1 expression based on the SP142-IHC assay was not a significant factor in RFS (Supplementary Table 4; *P* = 0.078).

In the multivariate analyses of RFS, only positive PD-L1 expression based on the 22C3-IHC assay was a prognostic factor of decreased recurrence (Supplementary Table 4; HR 0.114, 95% CI 0.015–0.848, *P* = 0.034), and not SP263 (Supplementary Table 4; *P* = 0.511).

## Discussion

In an era where immune checkpoint inhibitors are being utilized for TNBC, we investigated the prognostic impact of three different PD-L1 antibodies, for which coupled immune checkpoint inhibitors were available, namely PD-L1 (SP142)-atezolizumab, PD-L1 (22C3)-pembrolizumab, and PD-L1 (SP263)-durvalumab. To evaluate the prognostic discriminatory power of each antibody, we established prediction models including a null model that did not include PD-L1 expression data for model development. PD-L1 expression based on the 22C3-IHC assay was consistently the most powerful prognostic factor. Furthermore, pre-existing prediction models showed improved discriminatory power when PD-L1 expression based on the 22C3-IHC assay was added as a parameter. In this study, we focused on the manner in which PD-L1 expression based on different antibody assays correlated with long-term survival outcome and clinicopathologic characteristics.

Comparative analyses of concordant and discordant rates for different PD-L1 antibodies have not been widely reported^14–16^. PD-L1 has been studied as a prognostic factor in breast cancer^17^, with positive prognostic results being consistently reported despite the use of different antibodies and scoring methods^18–20^. Recently, the IMpassion130 trial demonstrated improved outcome in advanced TNBC patients presenting with PD-L1-expressing tumors who were treated with the SP142 antibody and the immune checkpoint inhibitor atezolizumab^4^. In addition to the IMpassion130 trial, the KEYNOTE-522 clinical trial demonstrated an improved pathological complete response rate among early TNBC patients with PD-L1 (22C3)-expressing tumors after neoadjuvant treatment combined with pembrolizumab^5^.

PD-L1 IHC is usually performed after surgical resection, and an accurate PD-L1 IHC assay could be done in the substantial amount of tumor tissue due to the tumor heterogeneity. Kim et al., reported relatively good agreement between small biopsy samples and surgical specimens in the three commercial PD-L1 antibodies (concordance rates of 73%–96%, 65%–80%, and 72%–91% between 26, 20, and 46 paired samples in 22C3, SP142, and SP263 PD-L1 IHC assays, respectively)^21^. Additionally, several studies have published reliable agreement rates between paired small tumor samples and surgical specimens^22,23^. In breast cancer, all three assays demonstrated good correlation for IC score and the concordance rate was the highest at a 1% cutoff value^16^.

This study has several limitations. First, this was a retrospective study conducted by utilizing samples collected before the immune checkpoint inhibitor era; therefore, we could not assess the real effect of immune checkpoint inhibitor on PD-L1-positive patients, Furthermore, although PD-L1 expression based on the 22C3-IHC assay seemed to exert the strongest influence on prognostic power, the RFS and DFMS graphs also demonstrated separation for the two other antibodies. With a sufficiently large population size, PD-L1 expression based on the SP263-IHC assay, and especially that based on the SP142-IHC assay, may demonstrate a significant prognostic effect. Interestingly, positive PD-L1 expression based on the SP142-IHC assay exerted no effect on RFS and DMFS, whereas PD-L1 expression based on the 22C3-IHC assay was revealed to be an independent prognostic factor. These results might have been derived from the cohort composition, as the cohort was not only composed of patients with TNBC but comprised a mixture of cases with varied molecular subtypes. Particularly, PD-L1 expression based on the SP142-IHC assay only evaluated %IC within the tumor area including the peri-tumoral stroma. As most TMA cores are composed of the intra-tumoral area, the peri-tumoral immune cell (IC) might have not been included. Moreover, TIL are heterogeneously distributed; thus, underestimation of TIL and subsequent underestimation of PD-L1 expression might have occurred. Conversely, PD-L1 expression evaluated using 22C3 was determined using the CPS method, wherein the denominator equaled the total number of tumor cells, and the numerator equaled the number of cells that showed positive staining for PD-L1 expression using 22C3. Using this method, the positivity rate using 22C3 might have been largely differed compared to that observed using SP142. However, this aspect could not explain the improved prognostic outcome of patients whose tumor tissues stained positively for PD-L1 expression in assays using the 22C3 antibody, and higher AUC values in the survival model with PD-L1 expression based on the 22C3-IHC assay.

In conclusion, our findings may indicate that PD-L1 expression based on the 22C3-IHC assay is a more powerful discriminatory marker than that based on SP142- and SP263-IHC assays in breast cancer. Our findings warrant additional validation using large-scale studies.

## Methods

### Patients

This retrospective study was initiated by collecting tumor tissues from 316 patients who underwent primary curative surgery for breast cancer between September 1999 and June 2015 at the Gangnam Severance Hospital in Seoul, Korea. All patients were treated according to standard protocols. The following data were recorded: age at surgery, tumor size, lymph node status, HG, status of ER, status of PR, status of the human epidermal growth factor receptor-2 (HER2), LVI, Ki67 leveling index, status of PD-L1 according to each antibody, treatment modalities, and survival outcomes. Tumor HG was determined using the modified Scarff–Bloom–Richardson grading system. Anatomical tumor-node-metastasis (TNM) classification was based on the TNM staging system of the American Joint Committee on Cancer, 8th edition.

All procedures were performed in accordance with the 1964 Declaration of Helsinki and its later amendments or comparable ethical standards. The study protocol was approved by the institutional review board (IRB) of the Gangnam Severance Hospital (local IRB No. 3–2018-0067). The need for informed consent was waived under the approval of the IRB due to the retrospective design of the study.

### TMA construction, IHC staining, and interpretation of results

On hematoxylin and eosin-stained slides of tumors, a representative area was selected, and the corresponding spot was marked on the surface of the paraffin-embedded block. Using a hollow needle, the selected area was punched out and the resulting 2-mm tissue core was placed in a 10 × 5 recipient block. Each separate tissue core was assigned a unique TMA location number that was linked to a database including other clinicopathologic data.

As per methods previously described^24^, 3 µm-thick tissue sections were sliced and obtained from formalin-fixed paraffin-embedded TMA blocks. After performing deparaffinization with xylene and rehydration with alcohol graded solutions, IHC was performed using the Ventana Discovery XT Automated Slide Stainer (Ventana Medical System, Tucson, AZ, USA). Cell Conditioning 1 buffer (citrate buffer, pH 6.0; Ventana Medical System) was used for antigen retrieval. The slices were incubated with the primary antibodies against estrogen receptor (ER; 1:150, clone 6F11; Novocastra Laboratories, Ltd., Newcastle upon Tyne, UK), progesterone receptor (PR; 1:100; clone 16; Novocastra Laboratories Ltd.), HER2 (1:1500; polyclonal; DAKO, Glostrup, Denmark), PD-L1 (prediluted; clone SP142; Ventana Medical System), PD-L1 (prediluted; clone SP263; Ventana Medical System), and PD-L1 (1:50; clone 22C3; DAKO). The appropriate positive and negative controls were included.

### Molecular subtyping

Nuclear staining values ≥ 1% were considered indicative of ER and PR positivity^25^. HER2 staining was interpreted based on the 2018 American Society of Clinical Oncology/College of American Pathologists guidelines^26^. Only samples with strong and circumferential membranous HER2 immunoreactivity (3 +) were considered positive, whereas those with 0 and 1 + HER2 staining were considered negative. Cases with equivocal HER2 expression (2+) were further evaluated for HER-2 gene amplification via silver in situ hybridization (SISH). Breast cancer subcategorization was based on the results of IHC staining for ER, PR, HER2, as well as the SISH results for HER2. The specimens were categorized as follows: (i) Luminal/HER2-negative (ER- and/or PR-positive and HER2-negative); (ii) HER2-positive (HER2-positive regardless of the ER and PR statuses); (iii) TNBC (ER-, PR-, and HER2-negative).

### Interpretation of PD-L1 immunohistochemistry results

For evaluating PD-L1 expression based on the 22C3-IHC assay, the CPS was calculated by dividing the number of stained cells expressing PD-L1 (tumor cells, lymphocytes, and macrophages) with the total number of viable tumor cells, and by multiplying the quotient by 100. CPS ≥ 1 was considered as positive.

$$\mathrm{CPS score}=\frac{\mathrm{ Number of PD}-\mathrm{L}1\mathrm{ staining cells }\left(\mathrm{tumor cell},\mathrm{ lymphocytes},\mathrm{ macrophages}\right) * 100}{\mathrm{Total number of viable tumor cells}}$$

For PD-L1 expression based on the SP142-IHC assay, the intensity of tumor-infiltrating IC staining was examined. Immune cells present in the intra-tumoral and contiguous peri-tumoral stroma, including lymphocytes, macrophages, dendritic cells, and granulocytes, were evaluated. The % IC was determined as the proportion of tumor area exhibiting PD-L1 staining of any intensity. For deducing PD-L1 expression based on the SP263 IHC assay, staining of immune cells at any intensity was considered positive staining, and the total percentage of signal intensity was visually estimated to generate data on the PD-L1 expression level.

$$\mathrm{\%IC }=\frac{percentage area of PD-L1 positive immune cells * 100}{percentage area of tumor area}$$

Representative images are shown in Fig. 1.

### Statistical analysis

Recurrence-free survival (RFS) was defined as the period between the primary surgery and any case of recurrence (loco-regional and/or distant metastasis) of breast cancer, death occurring due to any cause, or event of the last follow-up. DMFS was defined as the period between the primary curative surgery and diagnosis of breast cancer-derived distant metastasis, death occurring due to any cause, or event of the last follow-up. The data of patients who did not exhibit relevant events were censored at the completion of follow-up.

Clinical characteristics were compared between PD-L1-negative and -positive groups assessed using each antibody. Continuous variables between the two groups were compared using the Student’s *t*-test or the Mann–Whitney test. Categorical variables were compared using the *Chi*-square test or Fisher’s exact test. Survival curves were generated using the Kaplan–Meier method and compared between the two groups using the log-rank test. Cox proportional hazard models were used to identify factors associated with survival outcome (RFS and DMFS). We applied the backward likelihood method (specifies the significance level for entering effects = 0.10 and removing effects = 0.05) in the Cox proportional hazard models.

Time-dependent receiver operating characteristic curves were generated to ascertain the identity of the PD-L1 antibody that presented with the most considerable contribution to the prognostic value. Furthermore, to investigate the additional prognostic power of PD-L1 expression based on the IHC assays performed using the three antibodies, we calculated the Harrel’s c-index for each Cox proportional hazard model^27^. This helped measure the concordance for time-to-event data, in which increasing values between 0.5 and 1.0 indicated improved prediction. For model comparison, the bootstrapping method was used with resampling 1,000 times. We also assessed the NRI and IDI to evaluate the improvement in discriminatory ability contributed by PD-L1 expression using each of the three antibodies when data were added to the survival model^28^. Significant improvement was recognized in the prediction model when NRI > 0 and IDI > 0.

Statistical analysis was performed using the IBM SPSS Statistics version 24 software (IBM Corp., Armonk, NY, USA). The threshold for statistical significance was set at *P* < 0.05, with a 95% confidence interval (CI) not including 1.

### Consent for publication

All authors have provided consent for publication.

### Ethics approval and consent to participate

All procedures performed in studies involving human participants were in accordance with the ethical standards of the institutional and/or national research committee and the 1964 Helsinki Declaration. The protocol was approved by the institutional review board (local IRB No. 3–2018-0067) of Gangnam Severance Hospital. The need for informed consent was waived under the approval of the IRB due to the retrospective design.

## Supplementary Information

Supplementary Information 1.

Supplementary Information 2.

Supplementary Information 3.

Supplementary Information 4.

## Data Availability

The datasets generated and analyzed during the present study will be available from the corresponding author upon request.

## Abbreviations

AUC: Area under curve
CI: Confidence interval
CPS: Combined positive score
DMFS: Distant metastasis-free survival
ER: Estrogen receptor
HER2: Human epidermal growth factor receptor-2
HG: Histological grade
HR: Hazard ratio
IC: Immune cell
IDI: Integrated discrimination improvement
IHC: Immunohistochemical
IRB: Institutional review board
LVI: Lympho-vascular invasion
NRI: Net reclassification index
PD-1: Programmed cell death proteins 1
PD-L1: Programmed death ligand 1
PR: Progesterone receptor
RFS: Recurrence-free survival
SISH: Silver in situ hybridization
TIL: Tumor-infiltrating lymphocytes
TMA: Tissue microarray
TNBC: Triple-negative breast cancer
