# Supplementary material for: PD-L1 expression evaluated by 22C3 antibody is a better prognostic marker than SP142/SP263 antibodies in breast cancer patients after resection
Source: Sci Rep. 2021 Oct 1;11:19555. doi: 10.1038/s41598-021-97250-2 (PMC8486819; doi:10.1038/s41598-021-97250-2)
Supplement: Supplementary file 1 — Supplementary Information 1. [file 41598_2021_97250_MOESM1_ESM.docx]

**Supplementary table 1. Cox proportional hazard analysis for distant metastasis free survival (DMFS)**

|  | **Univariate analysis** | | **Multivariate analysis** | |
| --- | --- | --- | --- | --- |
|  | **HR (95% CI)** | ***P-*value** | **HR (95% CI)** | ***P-*value** |
| **Age** | 0.978 (0.949-1.007) | 0.1377 |  |  |
| **HG** |  | 0.9579 |  |  |
| **Ⅰ, Ⅱ** | 1 |  |  |  |
| **Ⅲ** | 0.983 (0.515-1.876) |  |  |  |
| **LVI** |  | 0.4077 |  |  |
| **Negative** | 1 |  |  |  |
| **Positive** | 1.425 (0.616-3.299) |  |  |  |
| **Ki67** | 0.990 (0.970-1.010) | 0.3382 |  |  |
| **ER** |  | 0.698 |  |  |
| **Negative** | 1 |  |  |  |
| **Positive** | 0.867 (0.423-1.780) |  |  |  |
| **PR** |  | 0.714 |  |  |
| **Negative** | 1 |  |  |  |
| **Positive** | 0.870 (0.413-1.832) |  |  |  |
| **HER2** |  | 0.134 |  |  |
| **Negative** | 1 |  |  |  |
| **Positive** | 0.454 (0.161-1.276) |  |  |  |
| **Tumor size** |  | 0.0742 |  | 0.0728 |
| **≤2cm** | 1 |  | 1 |  |
| **>2cm** | 1.937 (0.937-4.004) |  | 1.943 (0.940-4.015) |  |
| **Lymph node metastasis** |  | 0.1825 |  |  |
| **Negative** | 1 |  |  |  |
| **Positive** | 1.561 (0.811-3.003) |  |  |  |
| **Chemotherapy** |  | 0.8028 |  |  |
| **Not done** | 1 |  |  |  |
| **Done** | 1.128 (0.439-2.896) |  |  |  |
| **Radiotherapy** |  | 0.135 |  |  |
| **Not done** | 1 |  |  |  |
| **Done** | 1.645 (0.856-3.159) |  |  |  |
| **22C3** |  | 0.0378 |  | 0.0376 |
| **Negative** | 1 |  | 1 |  |
| **Positive** | 0.122 (0.017-0.888) |  | 0.121 (0.017-0.886) |  |
| **SP142** |  | 0.2629 |  |  |
| **Negative** | 1 |  |  |  |
| **Positive** | 0.443 (0.106-1.843) |  |  |  |
| **SP263** |  | 0.1788 |  |  |
| **Negative** | 1 |  |  |  |
| **Positive** | 0.597 (0.282-1.266) |  |  |  |

HR, hazard ratio; CI, confidence interval; HG, histologic grade; LVI, lympho-vascular invasion, ; ER, estrogen receptor; PR, progesterone receptor; HER-2, human epidermal growth factor receptor-2

**Supplementary table 2. Evaluation of multivariate Cox proportional hazard model using Harrel’s *c*-index, NRI, IDI and time dependent AUC for DMFS**

|  | **Null model** |  | **Model 1** |  | **Model 2** |  | **Model 3** |  |
| --- | --- | --- | --- | --- | --- | --- | --- | --- |
|  | **HR (95% CI)** | ***P*-value** | **HR (95% CI)** | ***P*-value** | **HR (95% CI)** | ***P*-value** | **HR (95% CI)** | ***P*-value** |
| **Harrel’s c index** | 0.573 (0.522-0.624) | 0.005 | 0.633 (0.574-0.692) | <0.0001 | 0.584 (0.521-0.647) | 0.0087 | 0.601 (0.528-0.674) | 0.0063 |
| **NRI** | 1 |  | 0.135 (0.0001-0.213) | <0.0001 | 0.047 (-0.11-0.138) | 0.1239 | 0.074 (-0.063-0.238) | 0.2458 |
| **IDI** | 1 |  | 0.02 (0.0003-0.042) | 0.044 | 0.003 (-0.0005-0.018) | 0.1518 | 0.007 (-0.001-0.034) | 0.1299 |
| **Time dependent AUC** | 0.570 (0.519-0.621) | 0.0071 | 0.634 (0.579-0.689) | <0.0001 | 0.584 (0.527-0.641) | 0.0038 | 0.596 (0.527-0.665) | 0.0061 |

AUC, area under the curve; CI, confidence interval; HR, hazard ratio; IDI, integrated discrimination improvement; NRI, net reclassification index; RFS, recurrence-free survival

*Null model: including tumor size

**Model 1: Null model + 22C3

***Model 2: Null model + SP142

****Model 3: Null model + SP263

**Supplementary table 3. Comparison of patient and tumor characteristics, and PD-L1 status in patients with triple negative breast cancer (TNBC)**

|  | **22C3** |  |  | **SP142** |  |  | **SP263** |  |  |
| --- | --- | --- | --- | --- | --- | --- | --- | --- | --- |
|  | **Negative, n=108,** | **Positive, n=46,** |  | **Negative, n=133,** | **Positive, n=34,** |  | **Negative, n=80,** | **Positive, n=85,** |  |
|  | **N (%)** | **N (%)** | ***P-value*** | **N (%)** | **N (%)** | ***P-value*** | **N (%)** | **N (%)** | ***P-value*** |
| **Age (year, mean±SD)** | 49.55±11.60 | 50.48±11.01 | 0.644 | 50.69±11.37 | 49.29±13.08 | 0.536 | 50.59±11.43 | 50.53±11.95 | 0.975 |
| **Ki67 (%, mean±SD)** | 30.77±21.44 | 45.48±21.61 | <0.001 | 33.94±23.41 | 39.61±18.94 | 0.160 | 30.49±22.40 | 39.82±21.67 | 0.012 |
| **HG^a^** |  |  | 0.088 |  |  | 0.053 |  |  | 0.001 |
| **I, II** | 32 (29.6) | 8 (17.4) |  | 40 (30.1) | 5 (14.7) |  | 31 (38.8) | 14 (16.5) |  |
| **III** | 72 (66.7) | 38 (82.6) |  | 87 (65.4) | 29 (85.3) |  | 46 (57.5) | 68 (80) |  |
| **Missing** | 4 (3.7) | 0 |  | 6 (4.5) | 0 |  | 3 (3.8) | 3 (3.5) |  |
| **AJCC stage^#, a^** |  |  | 0.105 |  |  | 0.040 |  |  | 0.070 |
| **I** | 32 (29.6) | 18 (39.1) |  | 39 (29.3) | 18 (54.5) |  | 22 (27.5) | 36 (42.4) |  |
| **II** | 58 (53.7) | 27 (58.7) |  | 78 (58.6) | 14 (42.4) |  | 46 (57.5) | 42 (49.4) |  |
| **III** | 14 (13.0) | 1 (2.2) |  | 13 (9.8) | 1 (3) |  | 9 (11.3) | 6 (7.1) |  |
| **Missing** | 4 (3.7) | 0 |  | 3 (2.3) | 0 |  | 3 (3.8) | 1 (1.2) |  |
| **LVI^a^** |  |  | 0.078 |  |  | 0.508 |  |  | 0.459 |
| **Negative** | 76 (70.4) | 39 (84.8) |  | 101 (75.9) | 26 (76.5) |  | 61 (76.3) | 65 (76.5) |  |
| **Positive** | 21 (19.4) | 4 (8.7) |  | 18 (13.5) | 3 (8.8) |  | 12 (15) | 9 (10.6) |  |
| **Missing** | 11 (10.2) | 3 (6.5) |  | 14 (10.5) | 5 (14.7) |  | 7 (8.8) | 11 (12.9) |  |
| **Chemotherapy** |  |  | 0.911 |  |  | 0.255 |  |  | 0.690 |
| **Done** | 98 (90.7) | 42 (91.3) |  | 122 (91.7) | 29 (85.3) |  | 73 (91.3) | 76 (89.4) |  |
| **Undone** | 10 (9.3) | 4 (8.7) |  | 11 (8.3) | 5 (14.7) |  | 7 (8.8) | 9 (10.6) |  |
| **Radiotherapy** |  |  | 0.569 |  |  | 0.249 |  |  | 0.870 |
| **Done** | 58 (53.7) | 27 (58.7) |  | 81 (60.9) | 17 (50) |  | 47 (58.8) | 51 (60) |  |
| **Undone** | 50 (46.3) | 19 (41.3) |  | 52 (39.1) | 17 (50) |  | 33 (41.3) | 34 (40) |  |

SD, standard deviation; HG, histological grade; LVI, lympho-vascular invasion

^a^Percentages calculated without missing values

**Supplementary table 4. Cox proportional hazard analysis for recurrence free survival (RFS) in TNBC**

|  | **Univariate analysis** | | **Multivariate analysis** | |
| --- | --- | --- | --- | --- |
|  | **HR (95% CI)** | ***P-*value** | **HR (95% CI)** | ***P-*value** |
| **Age** | 0.965 (0.930-1.002) | 0.065 |  |  |
| **HG** |  | 0.656 |  |  |
| **Ⅰ, Ⅱ** | 1 |  |  |  |
| **Ⅲ** | 0.914 (0.614-1.359) |  |  |  |
| **LVI** |  | 0.833 |  |  |
| **Negative** | 1 |  |  |  |
| **Positive** | 1.122 (0.385-3.273) |  |  |  |
| **Ki67** | 0.985 (0.963-1.007) | 0.181 |  |  |
| **Tumor size** |  | 0.089 |  |  |
| **≤2cm** | 1 |  |  |  |
| **>2cm** | 2.101 (0.892-4.946) |  |  |  |
| **Lymph node metastasis** |  | 0.001 |  | 0.074 |
| **Negative** | 1 |  | 1 |  |
| **Positive** | 3.477 (1.612-7.498) |  | 2.084 (0.930-4.668) |  |
| **Chemotherapy** |  | 0.454 |  |  |
| **Not done** | 1 |  |  |  |
| **Done** | 2.144 (0.291-15.786) |  |  |  |
| **Radiotherapy** |  | 0.883 |  |  |
| **Not done** | 1 |  |  |  |
| **Done** | 1.059 (0.495-2.262) |  |  |  |
| **22C3** |  | 0.021 |  | 0.034 |
| **Negative** | 1 |  | 1 |  |
| **Positive** | 0.095 (0.013-0.700) |  | 0.114 (0.015-0.848) |  |
| **SP142** |  | 0.078 |  |  |
| **Negative** | 1 |  |  |  |
| **Positive** | 0.166 (0.023-1.223) |  |  |  |
| **SP263** |  | 0.034 |  | 0.511 |
| **Negative** | 1 |  | 1 |  |
| **Positive** | 0.406 (0.176-0.934) |  | 0.721 (0.272-1.910) |  |

HR, hazard ratio; CI, confidence interval; HG, histologic grade; LVI, lympho-vascular invasion, ; ER, estrogen receptor; PR, progesterone receptor; HER-2, human epidermal growth factor receptor-2
