# Supplementary material for: PD-L1 expression evaluated by 22C3 antibody is a better prognostic marker than SP142/SP263 antibodies in breast cancer patients after resection
Source: Sci Rep. 2021 Oct 1;11:19555. doi: 10.1038/s41598-021-97250-2 (PMC8486819; doi:10.1038/s41598-021-97250-2)
Supplement: Supplementary file 2 — Supplementary Information 2. [file 41598_2021_97250_MOESM2_ESM.docx]

**Supplementary Figure 1.** Kaplan-Meier survival curves of recurrence-free survival (RFS) in relation to PD-L1 expression based on PD-L1 antibody-IHC assays in patients with triple-negative breast cancer. Negative PD-L1 expression was significantly associated with poor RFS with 22C3 (a), SP142 (b), and SP263 (c) antibodies

**Supplementary Figure 2.** Kaplan-Meier survival curves of distant metastasis-free survival (DMFS) in relation to PD-L1 expression based on PD-L1 antibody-IHC assays in patients with triple-negative breast cancer. (a) Patients with negative PD-L1 expression based on the 22C3-IHC assay had poor DMFS (HR 4.184, 95% CI 1.710-10.24, *P=*0.0017, log-rank test). (b) DMFS did not significantly differ based on SP142-IHC assay results. (c) PD-L1 negativity based on the SP263 IHC assay showed poor DMFS (HR 2.746, 95% CI 1.188-6.350, *P=*0.0181, log-rank test)
