# Supplementary figures and images for: PD-L1 expression evaluated by 22C3 antibody is a better prognostic marker than SP142/SP263 antibodies in breast cancer patients after resection
Source: Sci Rep. 2021 Oct 1;11:19555. doi: 10.1038/s41598-021-97250-2 (PMC8486819; doi:10.1038/s41598-021-97250-2)

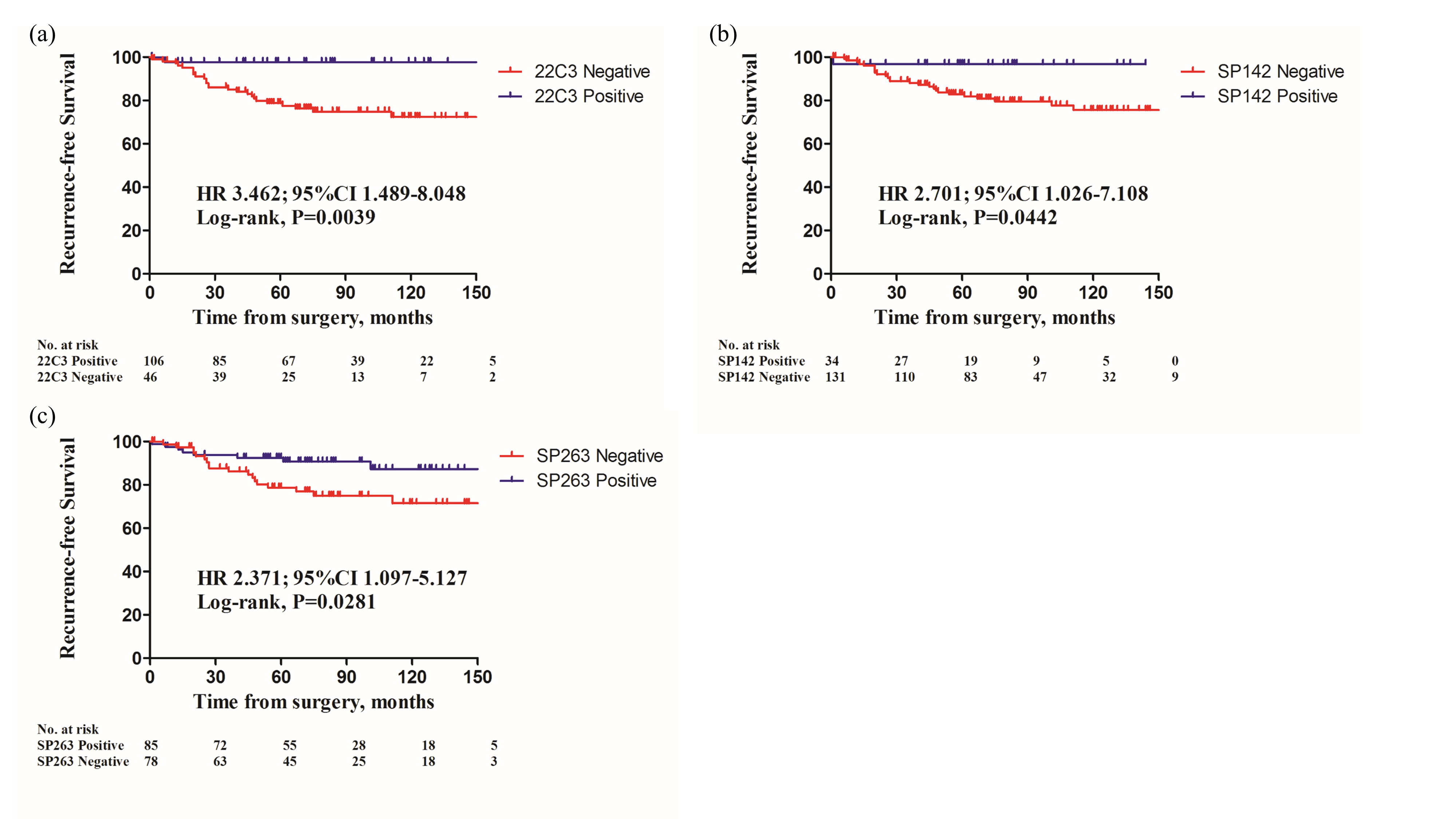

Supplement: Supplementary file 3 — Supplementary Information 3. [file 41598_2021_97250_MOESM3_ESM.tif]

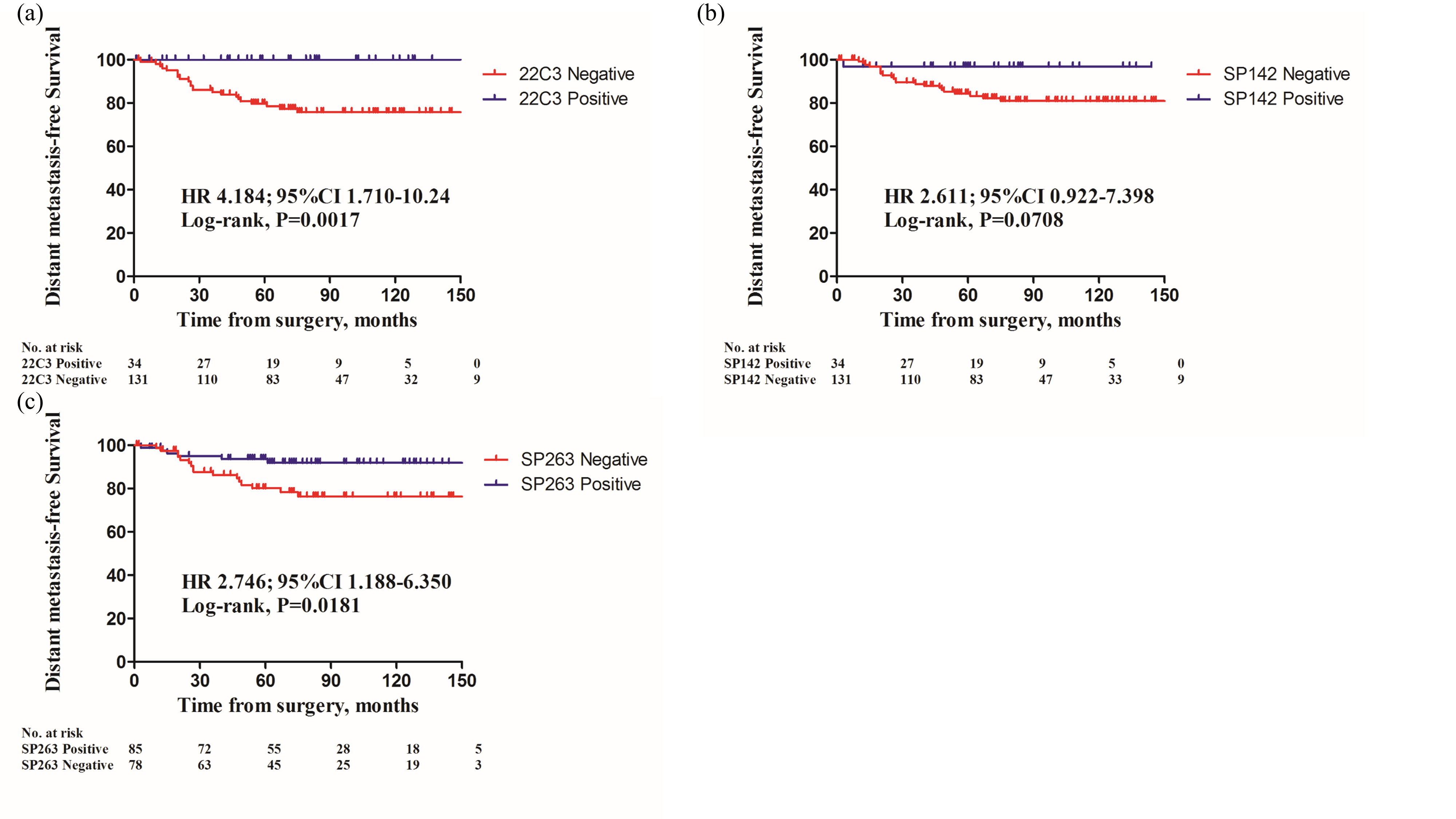

Supplement: Supplementary file 4 — Supplementary Information 4. [file 41598_2021_97250_MOESM4_ESM.tif]
